# Supplementary material for: Extracellular riboflavin induces anaerobic biofilm formation in Shewanella oneidensis
Source: Biotechnol Biofuels. 2021 Jun 3;14:130. doi: 10.1186/s13068-021-01981-3 (PMC8176591; doi:10.1186/s13068-021-01981-3)
Supplement: Supplementary file 1 — Additional file 1: Table S1. Strains used in this study. Table S2. Primers used in this study. Table S3. Parameters for simulation of S. oneidensis growth and riboflavin production both in batch and as continuous biofilm cultivation. Fig.S1. Impact of putrescine addition on current generation and biofilm formation on anode surfaces. Fig. S2. Impact of produced riboflavin on speC expression during growth. After 600 min 39 nM riboflavin are produced. As can be seen the relative speC expression doubles after 600 min. Fig. S3. Impact of speF deletion on current density. As can be seen the current density decreases significantly as a result of speF deletion. Fig. S4. Simulation results for a) biofilm thickness (front, middle and rear of the flow cell), b) lactate, c) FMN and d) riboflavin concentration. e) Simulated distribution of FMN and riboflavin over the average biofilm thickness after 140 h. [file 13068_2021_1981_MOESM1_ESM.pdf]

Supplements

Table S1: Strains used in this study.

Strain	Source
S. oneidensis wildtype + pBAD empty	This study
S. oneidensis Δ*speC* + pBAD empty	This study
S. oneidensis wildtype + pBAD *speC*	This study
S. oneidensis Δ*speC* + pBAD *speC*	This study
S. oneidensis Δ *srtA* + pBAD empty	This study
E. coli WM3064	W. Metcalf, Univ. of Illinois, Urbana
E. coli WM3064 + pMQ500up_500down_*speC*	This study
E. coli WM3064 + pMQ500up_500down_*speF*	This study
S. oneidensis Δ*speF*	This study

Table S2: Primers used in this study.

Num.	Name	Sequence	purpose
1	*speC*_OL_pBAD_for	GTTTAACTTTAAGAAGGAGATATACATACCATGAGCCAATTTCAATCTATTG	Construction of pBAD *speC*
2	*speC*_OL_pBAD_rev	CCGCCAAAACAGCCAAGCTGGAGACCGTTTTTACAGATAGTAATCTTTCAGT	Construction of pBAD *speC*
3	pBAD_test_for	GATTAGCGGATCCTACCTGAC	Sequencing of pBAD inserts *speC* and *srtA*
4	pBAD_test_rev	CTCTCATCCGCCAAAACAGC	Sequencing of pBAD inserts *speC* and *srtA*
5	500up_*speC*_OL_pMQ150_for	GATTACGAATTCGAGCTCGGTACCCGGGGATCTACTTCGACTCCAGTGAAGC	Construction of pMQ150 500up 500 down *speC*
6	500up_*speC*_OL_500down_*speC*_rev	CTTTTTGTTGCCATTAAGCCAATGGGCCAGAGTCTCCTTTG	Construction of pMQ150 500up 500 down *speC*
7	500down_*speC*_for	TTGGCTTAATGGCAACAAAAAG	Construction of pMQ150 500up 500 down *speC*
8	500down_*speC*_OL_pMQ150_rev	CGGCCAGTGCCAAGCTTGCATGCCTGCAGGCGTTAACAGACCCAATGCGT	Construction of pMQ150 500up 500 down *speC*
9	500up_*srtA*_OL_pMQ150_for	CAGTGCCAAGCTTGCATGCCTGCAGGGCGAGCCTAAGGAGTTAGT	Construction of pMQ150 500up 500 down *srtA*
10	500up_*srtA*_rev	CGAGCTCATCAATGGCTAAG	Construction of pMQ150 500up 500 down *srtA*
11	500down_*srtA*_OL_500up_*srtA*_for	CATTGATGAGCTCGACGGACGATAACGCGGCAGA	Construction of pMQ150 500up 500 down *srtA*
12	500down_*srtA*_OL_pMQ150_rev	TTACGAATTCGAGCTCGGTACCCGGGGATCGACCGACGTAAAGACCAAC	Construction of pMQ150 500up 500 down *srtA*
13	Test_Δ*speC*_for	GCCTAAGCGTAATGCCGA	Sequencing of Δ*speC*
14	Test_Δ*speC*_rev	TGCAAGAATTACCCTTTGCG	Sequencing of Δ*speC*
15	Test_Δ*srtA*_for	CGGTGCTGACCGATCTGCA	Sequencing of Δ*srtA*
16	Test_Δ*srtA*_rev	ACCAACTGGGCGATCAAGT	Sequencing of ΔsrtA
17	qPCR_*S.on*_for	TATTCAAGTGCTTCTATTAG	Cell quantification of *S. oneidensis* via qPCR
18	qPCR_*S.on*_rev	AAGAACTTCTACTCAACA	Cell quantification of *S. oneidensis* via qPCR
19	qPCR_*S.on*_*speC*_for	GTGAAGAGCATCTTCGACC	*S. oneidensis* *speC* quantfication via qPCR
20	qPCR_*S.on*_*speC*_rev	TAGTTAGCAGGGAAGCCG	*S. oneidensis* *speC* quantfication via qPCR
21	qPCR_*S.on*_*rpoA*_for	GAAGCTATCCGTCGTTCTGC	*S. oneidensis* *rpoA* quantfication via qPCR
22	qPCR_*S.on*_*rpoA*_rev	GACAGGACGCAACAGAATCG	*S. oneidensis* *rpoA* quantfication via qPCR
23	500up_*speF*_OL_pMQ150_for	GTCACGACGTTGTAAAACGACGGCCAGTGCCAAGCTTGCATGCCTGCAGGAAAGTTTGCGACTGTGGCTA	Construction of pMQ150 500up 500 down *speF*
24	500up_*speF*_rev	TTGAACCTGACTGCAGAGTTA	Construction of pMQ150 500up 500 down *speF*
25	500down_*speF*_OL_500up_*speF*_for	GAAATTGACTGCAGAGCGGGGTTATTAACTCTGCAGTCAGGTTCAACTAGTCGTTAATTTCCTTTTTACTTTAAAA	Construction of pMQ150 500up 500 down *speF*
26	500down_*speF*_OL_pMQ150_rev	GGAAACAGCTATGACCATGATTACGAATTCGAGCTCGGTACCCGGGGATCATCATATTTTTATTGATAACACAC	Construction of pMQ150 500up 500 down *speF*
27	Test_Δ*speF*_for	CAACCGATCAATGACACGC	Sequencing of Δ*speF*
28	Test_Δ*speF*_rev	CAGTCAATCATCCTTAAGTATC	Sequencing of Δ*speF*

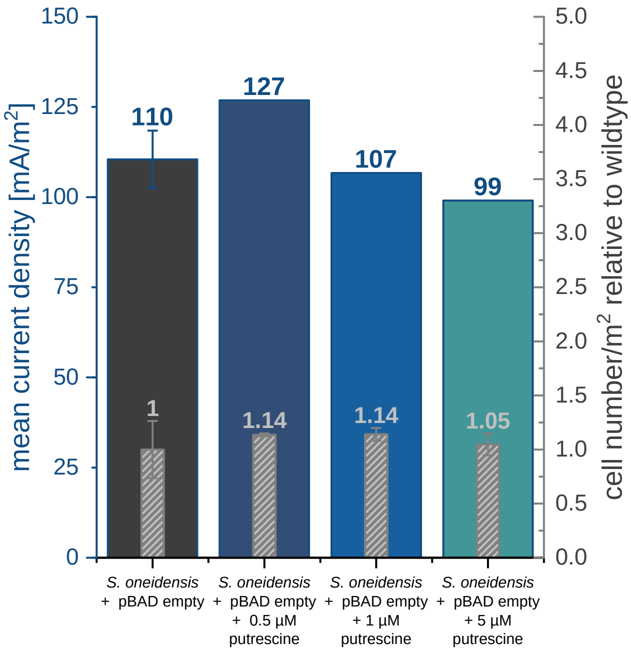

Fig.S1: Impact of putrescine addition on current generation and biofilm formation on anode surfaces.

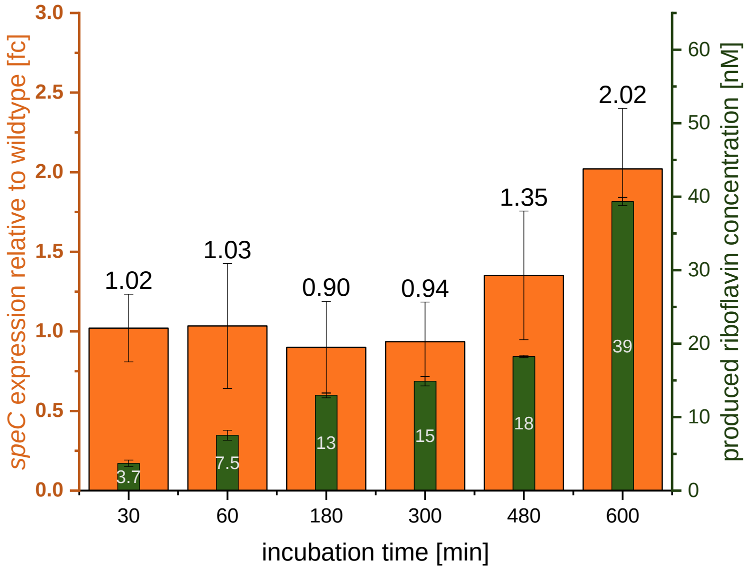

Fig. S2: Impact of produced riboflavin on *speC* expression during growth. After 600 min 39 nM riboflavin are produced. As can be seen the relative *speC* expression doubles after 600 min

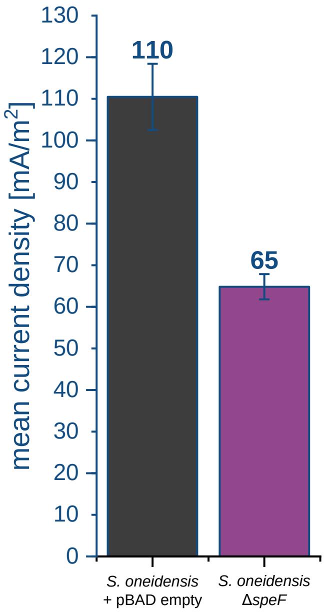

Fig. S3: Impact of *speF* deletion on current density. As can be seen the current density decreases significantly as a result of s*peF* deletion.

Batch and biofilm model

Based on the experimental results from batch and continuous biofilm cultivation a model was formulated to identify the driving factors for riboflavin production both in batch and biofilm systems. As can be seen from figure 4 the production of riboflavin is directly linked to growth of *S. oneidensis*. A Monod based double saturation approach (lactate and fumarate) was used to describe cell growth and FMN production. The transformation of FMN to riboflavin is then described with a first order reaction kinetic again coupled to the two Monod terms for lactate and fumarate.

$$\frac{\mathrm{dN}}{\mathrm{dt}} = \mu_{max} \frac{c_{Lac}}{K_S + c_{Lac}} \frac{c_{Fum}}{K_S + c_{Fum}} N \quad \left[\frac{cells}{m^3 h}\right] \quad \text{(S1)}$$

$$\frac{\mathrm{d}c_{Lac}}{\mathrm{dt}} = -\mu_{max} \frac{1}{Y_{cell/Lac}} \frac{c_{Lac}}{K_S + c_{Lac}} \frac{c_{Fum}}{K_S + c_{Fum}} N \quad \left[\frac{mol}{m^3 h}\right] \quad \text{(S2)}$$

$$\frac{\mathrm{d}c_{Fum}}{\mathrm{dt}} = -\mu_{max} \frac{1.3}{Y_{cell/Lac}} \frac{c_{Lac}}{K_S + c_{Lac}} \frac{c_{Fum}}{K_S + c_{Fum}} N \quad \left[\frac{mol}{m^3 h}\right] \quad \text{(S3)}$$

$$\frac{\mathrm{dP}_{FMN}}{\mathrm{dt}} = \mu_{max} Y_{FMN/cell} \frac{c_{Lac}}{K_S + c_{Lac}} \frac{c_{Fum}}{K_S + c_{Fum}} N \quad \left[\frac{\mu mol}{m^3 h}\right] \quad \text{(S4)}$$

$$\frac{\mathrm{dP}_{Ribo}}{\mathrm{dt}} = k_{Ribo} P_{FMN} \frac{c_{Lac}}{K_S + c_{Lac}} \frac{c_{Fum}}{K_S + c_{Fum}} \quad \left[\frac{\mu mol}{m^3 h}\right] \quad \text{(S5)}$$

N is the number of cells, μ_{max} the maximum growth at 30 °C, c_{Lac} the lactate concentration and c_{Fum} is the fumarate concentration (equation S1). $Y_{cell/Lac}$ is the yield coefficient for produced cells per transformed lactate (equations S2 and S3). P_{FMN} is the intermediate product (FMN) and $Y_{FMN/cell}$ is the growth related yield coefficient (equation S4). The latter is a Luedeking-Piret approach with growth related production of FMN (1). S5 is the first order reaction rate coupled to substrate availability. k_{Ribo} is the respective reaction rate constant.

In table S3 the kinetic and stoichiometric parameters are shown both for the batch culture and the biofilm simulation.

Table S3: Parameters for simulation of *S. oneidensis* growth and riboflavin production both in batch and as continuous biofilm cultivation

Parameter			Value batch experiments	Values for cultivation as biofilm
Growth rate	μ_{max}	h^{-1}	0.35	0.34
Monod constant	K_S	$mol\ m^{-3}$	22	25 – 120
Yield coefficient	$Y_{cell/Lac}$	$cells\ mol^{-1}$	1.3×10^{13}	7.0×10^{11}
Product yield	$Y_{FMN/cell}$	$\mu mol\ cell^{-1}$	9.0×10^{-13}	3.9×10^{-11}
FMN → Ribo	k_{Ribo}	h^{-1}	0.14	120
Cell density		$cells\ m^{-3}$	8.0×10^{14}	5.15×10^{16}
Diffusion coefficient:				
Riboflavin	D_{Ribo}	$m^2\ h^{-1}$	-	3.0×10^{-9}
FMN	D_{FMN}	$m^2\ h^{-1}$	-	1.9×10^{-6}
Lactate	D_{Lac}	$m^2\ h^{-1}$	-	4.2×10^{-6}

Knowing the production of FMN and riboflavin in a batch culture the next step was the simulation of its production in a biofilm. A 1D model was used to describe both biofilm and product formation (2, 3). The model is based on diffusion coupled with reaction inside the biofilm. The simulation tool AQUASIM was used to solve the equations (Reichert, 1998). The equations for substrate utilization and product formation inside the biofilm are then

$$\frac{\partial c_{Lac}}{\partial t} = -D_{Lac}\frac{\partial^2 c_{Lac}}{\partial z^2} - \mu_{max}\frac{1}{Y_{cell/Lac}}\frac{c_{Lac}}{K_S + c_{Lac}} N \qquad \left[\frac{mol}{m^3 h}\right] \qquad (S6)$$

$$\frac{\partial P_{FMN}}{\partial t} = -D_{FMN}\frac{\partial^2 P_{FMN}}{\partial z^2} + \mu_{max}\,Y_{FMN/cell}\,\frac{c_{Lac}}{K_S + c_{Lac}}\;N \quad \left[\frac{\mu mol}{m^3 h}\right] \tag{S7}$$

$$\frac{\partial P_{Ribo}}{\partial t} = -D_{Ribo}\frac{\partial^2 P_{Ribo}}{\partial z^2} + k_{Ribo}\,P_{FMN}\,\frac{c_{Lac}}{K_S + c_{Lac}} \quad \left[\frac{\mu mol}{m^3 h}\right] \tag{S8}$$

z is the vertical axis within the biofilm and *D* the diffusion coefficient, which can be calculated with the Wilke-Chang equation (4). However, the most sensitive parameters for the simulation of biofilm dynamics is, beside growth rate μ_{max} and yield coefficients (here $Y_{cell/lac}$ and $Y_{FMN/cell}$), the cell density within the biofilm (5). The latter is responsible on how quick the thickness of the biofilm will develop, or how much new grown cells do fit into a certain biofilm volume (5). In our case the density has been measured and could be introduced into the model (Fig. S3, Tab. S3). To describe the gradient along the flow cell used for biofilm cultivation, we divided available reactor volume and biofilm surface in three connected compartments (front, middle and rear).

It has already been shown that the release of product from aggregated cells is not always based on a simple diffusion process as formulated in equations S4 and S5. Partly products are fixed to the outer cell wall and can be released by pH modification Coles & Gross 1967 (6). The diffusion coefficient has been used as tool to fit the measured data of product release with a simulation (7). Here the diffusion coefficient D_{Ribo} used for riboflavin (see Tab. S3) is three orders of magnitude smaller compared to the value calculated with the Wilke-Chang equation.

The simulation results in figure S3 show a good fit for biofilm thickness L_F along the flow cell. Due to the small diameter of the flow cell and respective shear forces acting on the biofilm, detachment was assumed to depend on a maximum achievable biofilm thickness L_{F_max} (150 µm):

$$\frac{dL_F}{dt} = u_F * \frac{L_F}{L_{F\,max}} \tag{S9}$$

u_F is the velocity by which the biofilm surface is moving perpendicular to the substratum (2). The lactate removal has been simulated by mimicking the reduced availability of an electron acceptor (oxygen) with an increasing K_S value (Fig. S3 b).

Both FMN and riboflavin release are not properly displayed with the model approach (Fig. S3 c and d). Although the maximum concentration in the bulk can be reproduced, the combination of FMN production, its transformation to riboflavin and the adsorption/release process within the biofilm cannot sufficiently be simulated over time. A significant difference of the kinetic values chosen for batch and biofilm cultivation can be seen for k_{Ribo}. Whereas the value for batch cultivation is 0.14 1/d the riboflavin production within the biofilm can only be displayed with a k_{Ribo} 120 1/d. The main reason is the two times higher cell density (Tab. S3).

The high diffusion coefficient chosen for riboflavin helps to fit the final concentration values measured both for FMN and riboflavin within the biofilm (Fig. S3 e). However, the maximum concentrations might be misleading. Finally, the amount of riboflavin, which is fixed in the biofilm grown in the flow cell after 140 h is only **4.6×10^{-3} nmol**. On the other side the amount of riboflavin transported out of the system with the volumetric flow is about **24 nmol**.

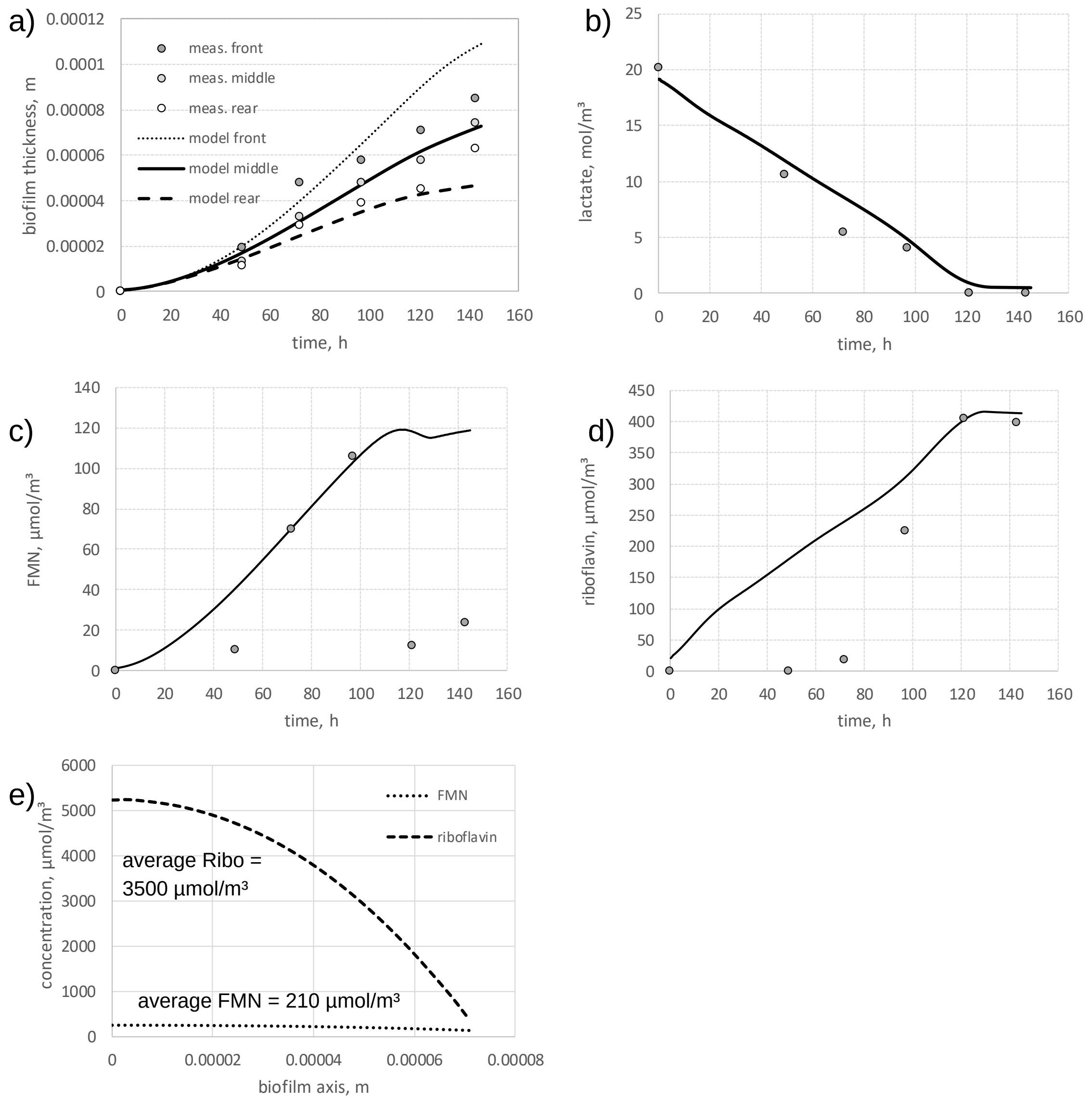

Fig. S4: Simulation results for a) biofilm thickness (front, middle and rear of the flow cell), b) lactate, c) FMN and d) riboflavin concentration. e) Simulated distribution of FMN and riboflavin over the average biofilm thickness after 140 h.

Literature

S1. Luedeking, R., (1967) Fermentation Process Kinetics, p. 181 - 243. Acadamic Press, London.

S2. Wanner, O., and Gujer, W. (1986) A Multispecies Biofilm Model. Biotechnology and Bioengineering 28: 314-328.

S3. Reichert, P., (1998) AQUASIM - Computer Program for Simulation and Data Analysis of Aquatic Systems. Schriftenreihe der EAWAG, Dübendorf.

S4. Wilke, C.R., and Chang, P. (1955) Correlation of diffusion coefficients in dilute solutions. AIChE Journal 1: 264-270.

S5. Horn, H., and Lackner, S. (2014) Modeling of Biofilm Systems: A Review. Advances in Biochemical Engineering/Biotechnology 146: 53-76.

S6. Coles, N.W. and Gross, R. (1967) Liberation of surface-located penicillinase from Staphylococcus aureus. Biochemical Journal 102(3), 742-747.

S7. Rinas, U., El-Enshasy, H., Emmler, M., Hille, A., Hempel, D.C., and Horn, H. (2005) Model-based prediction of substrate conversion and protein synthesis and excretion in recombinant *Aspergillus niger* biopellets. Chem. Sci. Eng. 60: 2729 – 2739.
